# Objective Evaluation of a New Epidural Simulator by the CompuFlo^®^ Epidural Instrument

**DOI:** 10.1155/2018/4710263

**Published:** 2018-06-26

**Authors:** Giorgio Capogna, Alessandra Coccoluto, Emanuele Capogna, Angelica Del Vecchio

**Affiliations:** ^1^European School of Obstetric Anesthesia (EESOA), Maternal Neonatal Simulation Centre, Roma, Italy; ^2^Policlinico Casilino Hospital, Roma, Italy

## Abstract

**Background:**

In this study, we describe a custom-made new epidural simulator, created by modifying the inner structure of a commercially available one, in the attempt to make it adequately realistic. To validate and evaluate the realism of our device, we used the Computerized Epidural Instrument CompuFlo.

**Method:**

The Compuflo CompuFlo curves obtained from 64 experiments on the epidural simulator were compared to 64 curves obtained from a previous human study, from consecutive laboring parturients requesting epidural analgesia.

**Results:**

Epidural simulator and human pressure curves were very similar. There was a significant difference between the drop of pressure due to false and true loss of resistance (LOR) in both the groups.

**Discussion:**

Our simulator can realistically reproduce the anatomical layers the needle must pass as demonstrated by the similarity between the simulator and human pressure curves and the small differences of pressure values recorded. CompuFlo may be used as an objective tool to create and assess and compare objectively the epidural task trainers.

## 1. Introduction

Manikin epidural simulators are based upon dummy models made from plastic or rubber. They are portable and easy to set up and use. One major limitation of these models is that they do not reproduce the anatomical features of all the layers the needle must pass (skin, subcutaneous fat, supraspinous and interspinous ligament, and ligamenta flava) and sometimes the solid plastic structure to represent the vertebare do not accurately reproduce the vertebral arch and the other possible bony parts which may be encountetred during needle insertion. The average impression of the users of the commercially available simulators, based on subjective scales, has been reported to be largely variable, usually achieving medium or low scores [1, 2].

In one recent study, four commercially available different epidural puncture training simulators were evaluated in a single-blinded, randomised controlled manikin study by expert consultant anesthesiologists, and none of the simulators tested reached a high enough score to indicate that they provided an experience close to a real patient [3].

In this study, we describe a custom-made new epidural simulator, created by modifying the inner structure of a commercially available one, and using some materials of our own design in the attempt to make it adequately realistic. The aim of the study was to validate and evaluate the realism of our device by using a Computerized Epidural Instrument (CompuFlo) [4].

## 2. Methods

**Figure 1:**
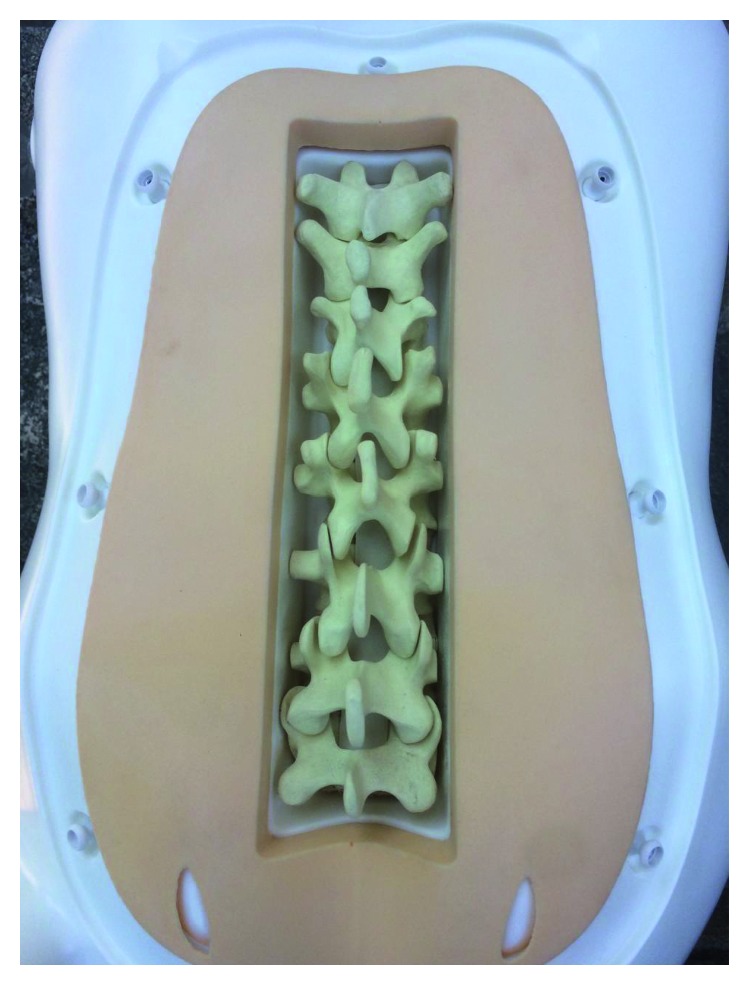
After having removed the inner length of plastic of a commercially available epidural simulator (P61, 3B Scientific), we inserted in its place a modified solid rigid polyurethane foam spinal column model. This way the vertebral arch and the other possible bony parts which may be encountered during needle insertion were present into the model in a life-like dimension and structure.

After having removed the inner length of plastic of a commercially available epidural simulator (P61, 3B Scientific, 3B Italy, Ozzano Bologna), we inserted in its place a modified solid rigid polyurethane foam spinal column model (SKU #1323-3, Sawbones, Europe). The spinal column model was sawn to obtain vertebral segments from L5 to T11. Each vertebra was modified by cutting the transverse processes and the dorsal part of the model in order to adapt it to the inside of the plastic torso. This way the vertebral arch and the other possible bony parts which may be encountered during needle insertion were present into the model in a life-like dimension and structure (Figure 1).

**Figure 2:**
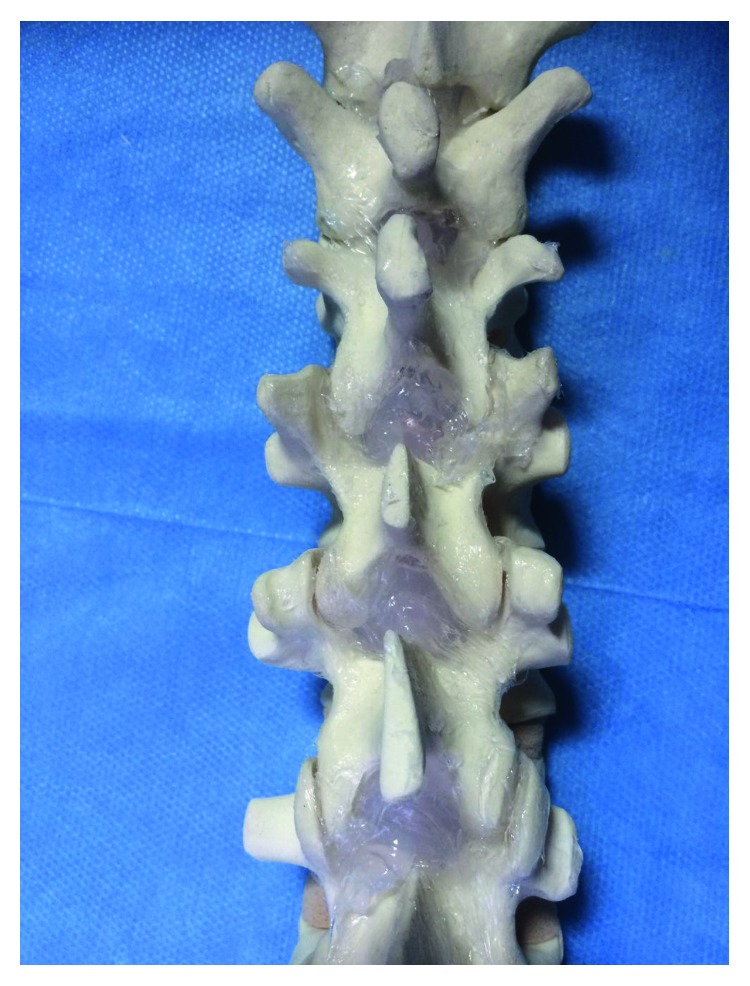
A layer of clear, transparent sealant silicone was modelled from the inferior edge and anteroinferior surface of cranial vertebra to the superior edge and posterosuperior surface of caudal vertebra to mimic the ligamenta flava.

A layer of clear, transparent sealant silicone (Bostik, UHU Bostik, Milano, Italy) was modelled from the inferior edge and anteroinferior surface of cranial vertebra to the superior edge and posterosuperior surface of caudal vertebra to mimic the ligamenta flava (Figure 2).

The silicone rubber exhibits tactile sensation similar to that of ligamentum flavum, providing hard texture for needle insertion and gives a tactile sensation when penetrated [5].

A standard closed-cell neoprene pipe insulation tube (Cestaro, Padova, Italy) has been reduced to pieces of the size, on the average, of 1 cm of diameter and mixed with the transparent sealant silicone in a ratio of 3 : 1 to mimic the ligamentum sovraspinosus. The same neoprene-silicone mixture was used for ligamentum interspinous and paravertebral muscles (multifidus), but in this case, the average diameter of the neoprene pieces was 1.5–2 cm in order to obtain a less-dense material capable to be crossed by the needle in a low resistant fashion.

**Figure 3:**
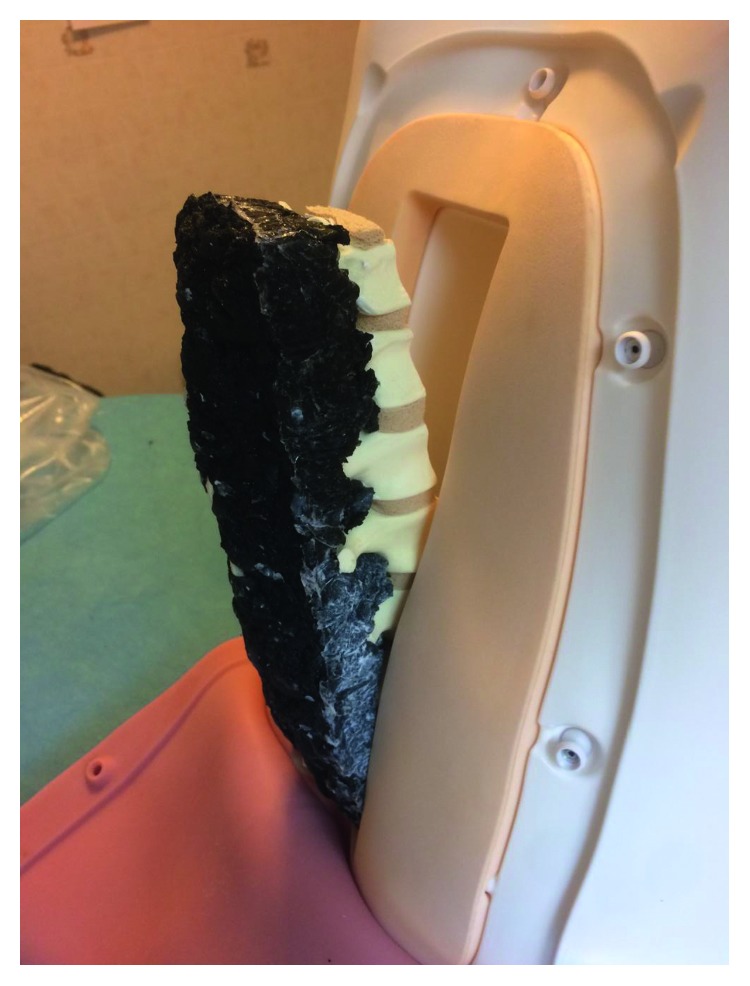
The neoprene-silicone mixture used for ligamentum interspinosus and paravertebral muscles (multifidus).

The dough taken was molded on the spine model to replace ligaments and muscles (Figure 3).

We completed the set with the standard artificial skin and the tubing and connectors representing the fluid-filled dural sac originally provided by the manufacturer (P61, 3B Scientific, Ozzano Bologna, Italy). This dura-simulating latex tube provides a distinct “pop” sensation when breached, indicating the tip of the needle has reached the subarachnoid space. When the stylet is withdrawn from the needle water which mimics “cerebrospinal fluid” that appears at the hub of the needle indicating the success of spinal puncture. The dura-simulating tube is self sealing allowing for repeated use.

We also modified the base body of the standard 3M Scientific simulator to better collect and drain the water used for loss of resistance to saline.

The CompuFlo Epidural Instrument (Milestone Scientific, Inc., Livingston, New Jersey, USA) [6] is a computer-controlled drug delivery system that can precisely measure the pressure of human tissues in real time at the tip of the needle. It is capable of distinguishing different tissue types by providing continuously real-time “exit-pressure” data at the needle tip when placed in-situ. The instrument uses an algorithm to determine the pressure at the tip of the needle via a continuous fluid path. This system is unique in that pressure is a feedback loop and controller to the system, thus regulating the electromechanical motor which controls flow-rate and the fluid dispensed by the system. An audible and visual graphic of exit-pressure is provided to the health care practitioner enabling the operator to focus on the injection site.

Using the CompuFlo, entry of the needle into the ligamentum flavum is indicated by a great increase in pressure with a simultaneous increase of the audible tone, while the entry of the needle into the epidural space results in a brisk drop in pressure on the visual display as well as a distinct fall in the tone of the audio output [4]. A drop in pressure sustained for more than 5 seconds is deemed consistent with entry into the epidural space. Typical curves can be obtained from epidural insertion, as reported in human studies [4, 7].

## 3. Experiment

For the purpose of the study, we used the same epidural technique and CompuFlo setting procedure previously used for one of our human studies [7].

The CompuFlo device was set to deliver normal saline at a rate of 0.05 mL/s with a maximum pressure limited to 200 mmHg, and the 0 point of pressure calibration was checked by positioning the needle at the level of the site of puncture.

In accordance with the standard anesthesia textbooks, the epidural needle (with stylet) should be placed into the ligamentum flavum before attaching the syringe, allowing an improved appreciation of epidural anatomy for the operator. However, in clinical practice, the epidural needle is frequently introduced in the lumbar area to a depth of approximately 2-3 cm to avoid accidental epidural space puncture. In this case, the needle may be inserted into the supraspinous or interspinous ligament, in the paraspinous muscles or subcutaneous tissue before the LOR with syringe is achieved, creating sometimes a pseudo or false LOR.

To evaluate both the LOR and the false LOR, we decided to introduce the epidural needle after skin puncture and to track the needle until the epidural space.

**Figure 4:**
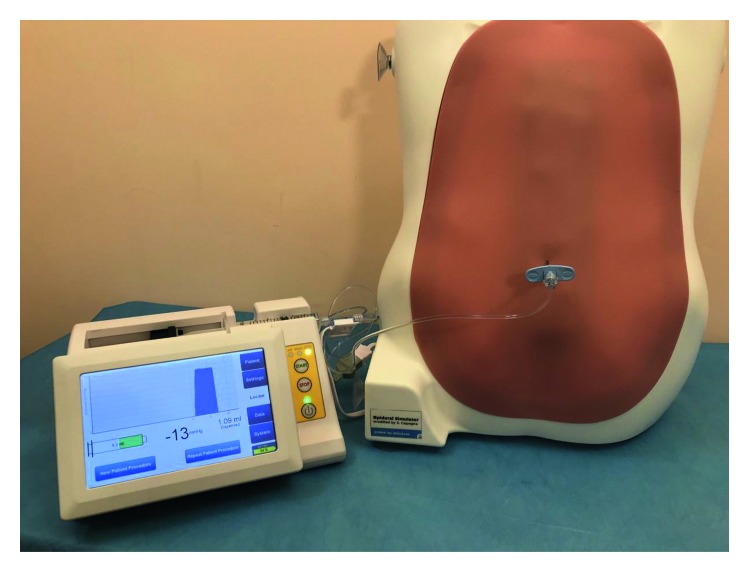
Connection of the CompuFlo instrument to the epidural simulator.

A Tuohy epidural needle 16G previously connected to the computerized injection pump via the arterial line extension tubing was introduced in the phantom and the CompuFlo instrument was started (Figure 4).

The Tuohy needle was then advanced very slowly, and pressures were recorded continuously.

**Figure 5:**
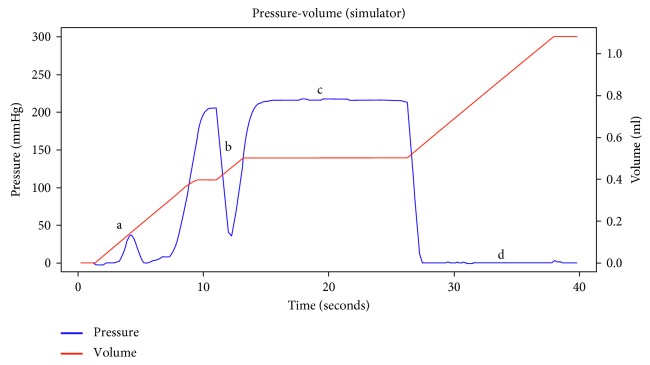
Typical pressure-volume infused curve during identification of the epidural space with Compuflo: a, supraspinous ligament (first small peak); b, false loss of resistance (first dip); c, legamentum flavum (increase in resistance); and d, final identification of the epidural space (sudden drop in pressure lasting for at least 5 seconds, resulting in the formation of a low and stable pressure plateau).

An increase in pressure followed by a sudden drop in pressure (typically greater than 50% of the maximum pressure) lasting for at least 5 seconds, resulting in the formation of a low and stable pressure plateau, was considered as definite epidural space identification (Figure 5).

If both these criteria were not fulfilled, and a small increase of delta of pressure followed by a transient drop of a lesser degree was observed, the result was considered as “false loss of resistance,” and the epidural needle was further advanced and/or repositioned until a true loss of resistance was obtained.

A sample size of 64 observations for each group was required to set 80% test power and a 95% significance level. The CompuFlo curves obtained from 64 experiments on the epidural simulator were compared to 64 curves obtained from a previous human study, from consecutive laboring parturients requesting epidural analgesia [7].

The differences between the mean of the two curves (the CompuFlo analysis in vivo and in simulation) were analyzed by using the R software. Differences in pressure drop were compared using unpaired two samples *t*-test.

## 4. Results

**Figure 6:**
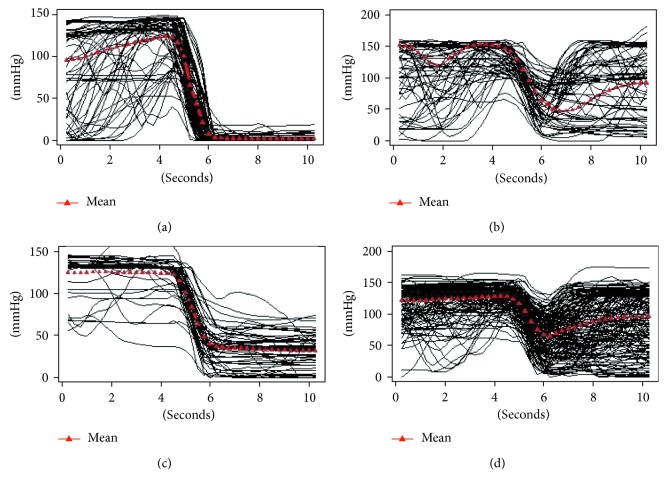
Epidural simulator and human pressure curves immediately before and after the needle have entered the epidural space. (a) LOR ES simulator, (b) false LOR simulator, (c) LOR ES, and (d) false LOR. LOR ES = loss of resistance in the epidural space, False LOR = false loss of resistance (needle not in the epidural space).

Epidural simulator and human pressure curves were very comparable (Figure 6).

As expected, we observed more variability in the plateau values indicating the epidural space in the control group as compared to those obtained with the simulator. This is obvious since epidural pressure is variable in humans, being approximately 15–20 mmHg in pregnancy, while the “epidural pressure” in the simulator is always equal to the atmospheric pressure, and comparable to that reported in nonpregnant subjects [8].

There was a significant difference in percentage of drop of pressure between the drop of pressure due to false loss of resistance (LOR) and that due to the entry of the needle in the epidural space in both the groups (Table 1).

## 5. Discussion

In this study, we reported the description of a new custom-made simulator and demonstrated its realism by the similarity between the simulator and human pressure curves objectively obtained with the CompuFlo Epidural instrument.

In a large review, Stunt et al. [9] reported that there are, on the average, more than 400 commercially available simulators for medical training, but 93.5% percent of these are not known to be tested for validity. The majority of studies on medical simulators did not describe a power analysis and lacked standardized assessment methods and blinded assessors.

To guarantee patient safety, it is important that simulators designed for each type of training demonstrate high levels of validity.

In particular, the major problems with epidural simulator assessment is that they are usually evaluated by subjective Likert like scales reported by physicians, and this kind of scales may have some disadvantages and are subjected to a large variability of ratings [1–3]. Due to the nature of this subjective evaluation, results of previous studies on epidural simulators are controversial.

In one study [2], the simulator was thought to be borderline for life-likeness for the “feel” of the supraspinous ligament and ligamentum flavum, and in another study, none of the commercially available simulators tested were considered to provide an experience close to a real patient [3].

The limits of the subjective physicians' evaluation are witnessed by one blinded study [3] that compared the identification of landmarks by palpation to select the point of puncture in different epidural simulators and reported that some participants felt that the palpation of landmarks with the banana were highly realistic, despite the fact that there were no vertebrae-like structures to feel.

To overcome such subjective nature of human evaluation, we decided to use the Computerized Epidural Instrument CompuFlo as an assessment tool to more impartially evaluate the realism of our simulator.

The results of this study suggest that our simulator can realistically reproduce the anatomical features of the layers the needle must pass through to reach the epidural space as demonstrated by the similarity between the simulator and human pressure curves.

Naemura et al. [10] studied the force of penetration waveforms of the epidural needle into a porcine model, reporting two peaks representing the needle penetrating in the supraspinous ligament and/or the muscle fiber and in the ligamentum flavum. Conventional simulators have been reported to be able to show only the simple increase up to peak when the needle is in the ligamentum flavum, followed by a plateau when the needle reaches the epidural space.

The biphasic curve observed in our simulator confirms the capability of our new device to reproduce even the subtle pressure changes due to the passage of the needle through the supraspinous ligamentum. In addition, it was able to identify the false loss of resistance due to the position of needle into the interspinous ligament, in the paraspinous muscles or in the subcutaneous tissue before the LOR with syringe is achieved. These features may positively contribute to improve the realism of the simulator.

We are certainly aware that our preliminary evaluation should be confirmed by clinical follow-up to investigate the training efficacy of such instrument in clinical teaching practice, and studies on this matter are in progress.

For correct medical psychomotor skills training and to provide objective and correct feedback, it is essential to have a realistic training environment. Scientific testing of simulators is an important way to prove and validate the training method. To guarantee effective training skills and patient safety, it is important that epidural simulators designed for this type of training demonstrate high levels of validity. We believe that the method we used to assess the realism of our simulator may be useful in future studies to better evaluate validity of a device as condition to guarantee proper acquisition of psychomotor skills.

In conclusion, our results suggest that our simulator can realistically reproduce the anatomical features of the layers the needle must pass through to reach the epidural space as demonstrated by the similarity between the simulator and human pressure curves and the small differences of pressure values recorded during the needle insertion. Our simulator may be a promising, realistic device in teaching epidural needle-handling skills for epidural technique. CompuFlo may be used as an objective tool to create and assess and compare objectively the epidural task trainers.

## Figures and Tables

**Table 1:** Differences in the mean values of false and true LOR (loss of resistance) between the drop of pressure observed with the simulator when compared to those of the controls.

Mean % of drop of pressure (mean, CI)	False LOR	True LOR (epidural space)	*P*
Simulator	9 (0.04–23)	97 (96–98)	0.04
Control	5.5 (2–13)	65 (63–75)	0.03

## Data Availability

The data used to support the findings of this study are available from the corresponding author upon request.
